# Loyalty to the past

**Published:** 1907-08-10

**Authors:** 


### Nursing Administration.

### LOYALTY TO THE PAST.

Every public office is held under conditions which bring forcibly to mind sooner or later the unimportance of the individual. The heads of institutions in particular are able to discern through all the weight of affairs which throws them into prominence that they are but units in a long series, and that the turn of events which has set them at the head is a call not so much to alter details and appropriate authority as to inherit and develop good work towards which many have contributed and to which many must still succeed. Regarded in the true historic spirit, the old blunders and makeshifts of the pioneers of nursing produce in the modern mind a humility bordering on reverence. What stupendous exertions went to the moulding of a good nurse in days not yet so very distant. What strain and stress devolved on the matron whose labours were unrelieved by a single modern appliance, whose nurses were tucked away in holes and corners, and whose domestic staff was summed up in a couple of charwomen. When the veil which covers the past is lifted, as may sometimes happen, the picture of hospital misrule which it is apt to disclose shows shining clearly through all the frequent disorders of imperfect organisation the figures of those whose triumphs were won under these adverse conditions, and whose character gained in force and beauty through the want of those very advantages which are now at the disposition of all. Rare qualities of head and heart were the dower of those matrons of old, and if the newer generation be ever tempted to a sense of easy superiority when they reflect upon the perfection of modern routine, they will do well to temper their satisfaction in their own methods with the wholesome question, "Under like circumstances, could I have done as well?" The matron who has set herself to realise that she is called to fill but a niche in a long gallery derives a wonderful sense of cheer from this reflection. She is no longer alone in her heavy cares and responsibilities. Others have laboured and sighed and triumphed in the same office, others will sit where she sits, turn over her memoranda, copy perhaps her regulations. The thought makes her very lenient to the mistakes of her predecessors, jealous of their reputation as of her own, eager to guard them from hasty condemnation. Even that most difficult of all relations, the relation of the matron towards her immediate predecessor, is rendered beautiful by such considerations. "I cannot condemn," said one who was suffering under the jealousy and misrepresentations of her successor, "for I alone am able to understand her difficulty." Hence in succeeding even a notoriously incompetent person, pity and oblivion are indicated, and the tendency to hark back to old grievances must be checked with a firm hand by the matron upon whom such ill-judged comparisons may seem to reflect credit. It does undoubtedly demand a vigorous effort to be strictly loyal to the immediate predecessor, however brilliant her talents. The unhappy drop of envy which lurks unsuspected in many otherwise noble natures makes it specially hard to follow a really popular matron, and the inclination to discount her successes by unveiling some instance of fallibility may need some self-disciplined effort to repress.

There is no field in which loyalty to the past is more fruitfully displayed than in the matron's attitude towards former members of the staff. The duty of welcoming visits and reports from nurses trained in the institution is comparatively easy so long as the same matron remains at the head of the training-school. Every matron takes pleasure in the successes of her pupils, and is naturally disposed to sympathise in their difficulties. But it is less easy for her to enter into the doings of strangers, and the inclination is to "let bygones be bygones." Yet the spirit of continuity in a good training-school is a thing worth some effort to preserve, and it is the duty of each matron in turn to take some trouble that it does not suffer at her hands. The sense that each worker is one of a long series dependent on predecessors for much that is making up the essence of the life lived under the shadow of the old walls, is never better tasted than in welcoming some unknown nurse back to the institution which has been a rallying call to her amidst countless difficulties in places where perhaps its name is altogether unknown. In this as in the right performance of all the fastidious details which go to make up her day's routine, the matron receives as much as she gives.

As the head *pro tem.* of the Hospital League or as hostess at the annual gatherings which bring former workers back from far and near, the matron may be brought into contact with nurses trained under more than one previous régime. If she has schooled herself to think that nothing was ever done properly until she came she will regard these visitors with indifference if not contempt. But if the matrons in the past have come to stand in her imagination as sisters to her in office, sisters whose faults she cannot help knowing, but whose memory she cherishes as coadjutors in the task of making and preserving the traditions of the institution, then she will welcome whatever serves to illustrate that continuity through which she herself will one day survive. Hopefulness about the future, that is the spirit engendered by loyalty to the past. No carping at change, no sinking of the heart when the day's work is done, and a successor all unused to the traditional methods is chosen to carry on the familiar loved duties. To live in the present with the consciousness fully alive that other hands will turn the registers so laboriously kept, that other eyes will scrutinise the arrangements made with such exactitude, is to engender a deep sense of responsibility which bears in the end rich fruit. It is not on gala days of compliment and congratulation that the matron's reputation is built up. It is through the hours overfilled with petty details during which sometimes there falls on the mind with refreshing balm a vision of accomplishment, when the long day shall be ended and the name inscribed among the number of those whose labours have tended to the glory of the hospital, and the greater honour of humanity at large.

